# Receipt of Weekly Iron Supplementation among Indian Children, 2005–2016

**DOI:** 10.1093/cdn/nzab020

**Published:** 2021-03-03

**Authors:** Rajesh Kumar Rai, Sabri Bromage, Wafaie W Fawzi

**Affiliations:** Society for Health and Demographic Surveillance, Suri, West Bengal, India; Department of Global Health and Population, Harvard TH Chan School of Public Health, Boston, MA, USA; Department of Economics, University of Göttingen, Göttingen, Germany; Centre for Modern Indian Studies, University of Göttingen, Göttingen, Germany; Department of Nutrition, Harvard TH Chan School of Public Health, Boston, MA, USA; Department of Global Health and Population, Harvard TH Chan School of Public Health, Boston, MA, USA; Department of Nutrition, Harvard TH Chan School of Public Health, Boston, MA, USA; Department of Epidemiology, Harvard TH Chan School of Public Health, Boston, MA, USA

**Keywords:** anemia, iron supplementation, iron deficiency, National Family Health Survey, India

## Abstract

**Background:**

In response to India's unacceptably high burden of anemia among children aged 6–59 mo, the central government introduced the National Iron Plus Initiative program which recommends an intervention of iron supplementation to mitigate anemia, especially iron deficiency anemia.

**Objective:**

The objective of this study was to examine the trend (between 2005–2006 and 2015–2016) in receiving weekly iron supplementation (WIS) among children aged 6–59 mo, and factors associated with receiving WIS during 2015–2016.

**Methods:**

Two waves of the nationally representative cross-sectional National Family Health Survey (NFHS) data collected during 2005–2006 (NFHS-3) and 2015–2016 (NFHS-4) were used. The trend was measured using both rounds of datasets, whereas factors associated with WIS receipt were assessed from NFHS-4. The trend was assessed using a sample of 35,650 children from NFHS-3 and 202,227 children from NFHS-4. After exclusion of 8978 cases, a total of 199,110 children were included to analyze the factors associated with receiving WIS. Using appropriate sample weighting, unadjusted and adjusted (multivariate) logistic regression analyses were deployed. Application of the chi-squared test and checking for multicollinearity were also part of the analysis. The possibility of sample selection bias was tested.

**Results:**

An increase of WIS receipt (from 4.6% in 2005–2006 to 26% in 2015–2016) was observed. Older children, children living in rural areas, children belonging to Scheduled Tribes, children of mothers with secondary education or higher, and children whose mothers had some mass media exposure had higher odds of receiving WIS. Children of fifth or higher birth order, children who were followers of Islam and Christianity, children from the richest economic group, noninstitutional birth of children, and children from high-focus group states were negatively associated with WIS receipt.

**Conclusions:**

Despite improvement (between 2005–2006 and 2015–2016) in receiving WIS, coverage remains unacceptably low (in absolute terms). The suboptimum performance of WIS intervention demands further investigation.

## Introduction

As of 2016, an estimated 58.7% of Indian children aged 6–59 mo suffer from anemia, defined as a hemoglobin concentration in blood below the established cut-off levels (1). This high burden of cases poses a daunting challenge not only to India's public health system from increased risk of child mortality, but due to the adverse consequences of iron deficiency anemia (IDA) on cognitive development, school performance, and physical development of children, and work productivity in adulthood (2–4). Anemia is multifactorial, and it is estimated that half of anemia cases are due to IDA (5), which could be treated with iron supplementation (4, 6).

To mitigate anemia among children, the Indian government launched the National Nutritional Anaemia Control Program in 1970 (7), and since then various efforts have been made to reduce the anemia burden with negligible success. The current prime minister of India has responded to the importance of reducing anemia with the introduction of the Anemia *Mukt Bharat* (literally “Anemia-Free India”) movement (8). According to the current guidelines of the National Iron Plus Initiative (NIPI) introduced in 2011 (9), iron-and-folic-acid (IFA) tablets or equivalent syrup should be given to individuals across various age groups of children and women in all states and union territories of India. All children aged 6–59 mo are to be given a 1 mL dose of IFA syrup containing 20 mg of elemental iron and 100 mcg of folic acid biweekly, along with biannual deworming for children aged 12 mo and above. Under NIPI, children aged 6–59 mo should be administered biweekly with IFA supplementation under direct supervision of the Accredited Social Health Activist (ASHA) workers on fixed days (recommended days are Monday and Thursday). NIPI is expected to benefit over 124 million children in India. Although the execution and effectiveness of NIPI have been questioned (7, 8, 10, 11), a recent report of the 2016–2018 Comprehensive National Nutrition Survey (CNNS) showed a reduction in anemia among children (12).

Though many studies and reports have examined factors associated with the burden of anemia among children (13–17), a serious dearth in research on determinants of receiving iron supplementation among children hinders devising an appropriate intervention. Existing studies (18, 19) on this issue either lack external validity or are outdated. Also, data to analyze the performance of the NIPI program are not available, which calls for exploring existing publicly available datasets. Against this evidence gap, using 2 consecutive waves of the nationally representative National Family Health Survey (NFHS), we assessed the trend (between 2005–2006 and 2015–2016) in weekly receipt of iron (tablet or syrup) supplementation (WIS) among children aged 6–59 mo, and factors associated with receiving the supplement in 2015–2016. An add-on analysis of understanding how the receipt of WIS differs with the anemia status of children in different states/union territories was also undertaken, along with an analysis of the burden of anemia and prevalence of WIS receipt in 640 districts of Indian during 2015–2016. Findings from this study are hopefully useful to understand the strengths and limitations of WIS and the ongoing NIPI program.

## Methods

### Dataset

Datasets used for this study were obtained from the third (2005–2006) (20) and fourth (2015–2016) (21) waves of the Demographic and Health Survey (DHS), popularly known as NFHS in India. NFHS is a cross-sectional survey, covering a nationally representative sample, and collects essential indicators on public health, demography, and vital components of clinical, anthropometric, and biochemical measures. NFHS was conducted under the stewardship of the Ministry of Health and Family Welfare, Government of India, and the International Institute for Population Sciences, Mumbai, India, was the nodal agency for conducting the survey. NFHS 2005–2006 (NFHS-3) and NFHS 2015–2016 (NFHS-4) used a sampling frame of India's 2001 Census and 2011 Census, respectively. NFHS-3 covered 124,385 women (aged 15–49 y) and 69,751 men (aged 15–54 y) living in 109,041 households, whereas NFHS-4 covered 699,686 women and 112,122 men of the same age group, and both surveys had over a 95% response rate. Further details about the NFHS sampling procedure can be found in its published reports (20, 21).

**FIGURE 1:**
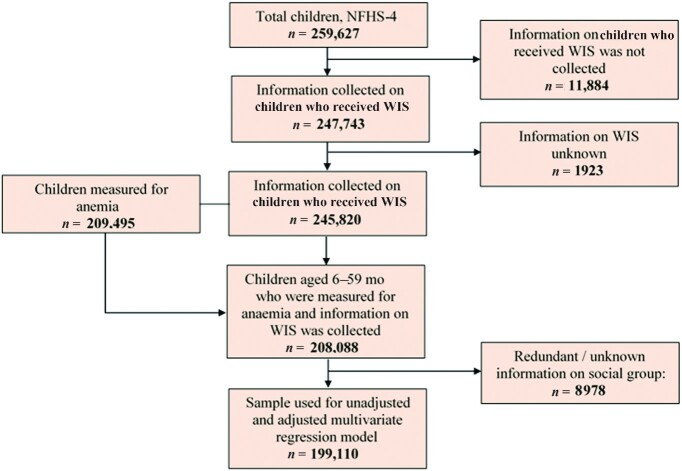
Flow chart on derivation of sample size. NFHS-4, National Family Health Survey 2015–2016; WIS, weekly iron supplementation.

Keeping with the study objective, this study included children who were aged 6–59 mo. Information on children was provided by their mothers, including information on WIS. To assess the trend of WIS receipt (between 2005–2006 and 2015–2016), a sample of 35,650 children (unweighted) from NFHS-3 and a sample of 202,227 children (unweighted) from NFHS-4 were compared, representing 28 states/union territories in both NFHS-3 and NFHS-4. To examine factors associated with WIS receipt, a sample of 199,110 children (unweighted) from NFHS-4 was analyzed (8978 children were excluded from the analysis due to unavailability of information on their social group). The derivation of the sample of 199,110 children is presented in **Figure 1**. As the exclusion of cases may lead to biased findings, the sample of 8978 children was compared with 199,110 children to rule out the possibility of sample selection bias. It is worth noting that NFHS-4 covered 37 states/union territories and 640 districts of India, but no district-level information was available in NFHS-3.

### Outcome measure

In both NFHS-3 and NFHS-4, women aged 15–49 y were asked about the history of receiving iron supplementation (tablet or syrup) for all their children born in the 5 y preceding the survey date. Thus, children born in 2001 or after and children born in 2011 or after were eligible to be included in the analysis from NFHS-3 and NFHS-4, respectively. In the NFHS, women were asked: “In the last 7 d, was (name of the child) given iron pills or iron syrup?” While asking the question, a sample of common iron tablets and iron syrups were shown to the respondent to reduce recall errors. The response was recorded as “yes,” “no,” or “don't know.” The responses against don't know were negligible (<1%), possibly because women only had to recall for the past 1 wk. No further information on WIS was collected. Information on receiving iron supplementation in the past 7 d from the survey date was defined as “weekly iron supplementation” or WIS in this study.

### Potential predictors

Guided by the existing literature on receipt of iron-and-folic-acid supplementation in India (10, 11, 18, 19, 22), a range of potential predictors was adopted to understand the factors associated with WIS receipt. The predictors and their subgroups (in parentheses) are age of children in years (0, 1, 2, 3, and 4), sex of child (male and female), child's birth order (first, second, third, fourth, and fifth or higher), areas of residence (urban and rural), religion (Hinduism, Islam, Christianity, Sikhism, and others), social group (others, Scheduled Castes, Scheduled Tribes [STs], and Other Backward Classes), wealth index (poorest, poorer, middle, richer, and richest), mother's age (in completed years) at birth of her children (<17, 17–19, 20–24, 25–29, and ≥30), mother's education (no or incomplete primary, primary or incomplete secondary, and secondary or higher), delivery care (institutional and noninstitutional), mass media exposure (no exposure and some exposure), states/union territories (high-focus group states/union territories and nonhigh-focus group states/union territories), and anemia status of children (anemic and nonanemic).

Principal component analysis was conducted to develop a wealth index using data on households' ownership of select assets, materials used for housing construction, and types of water access and sanitation facilities (23). Delivery of children that took place at private or public health institutions were considered an institutional delivery, whereas birth at home or other places were labeled as noninstitutional (24). The place of delivery is relevant because children born in a health facility may have a higher chance of receiving WIS. The NFHS asked women about their frequency of reading a newspaper or magazine, frequency of listening to a radio, and frequency of watching television on a weekly basis. Women having no engagement with any of these 3 forms of mass media were identified as having no exposure to mass media, otherwise they were classified as having some form of mass media exposure (25). On account of high fertility and high mortality indicators, 9 states are regarded as high-focus states in need of special attention: Bihar, Chhattisgarh, Jharkhand, Madhya Pradesh, Odisha, Rajasthan, Uttarakhand, Uttar Pradesh, and Assam (26). The Indian government runs various public health interventions targeting high-focus states, thus one may expect that children residing in high-focus states have higher odds of receiving WIS than in other states. Anemia status among children was considered to be one of the potential predictors of WIS receipt. In India, trained community health workers from the local public health facility are expected to test children for anemia and based on the diagnosis, children are expected to receive healthcare advice. Hence, one may also expect that anemic children have a higher probability of receiving WIS compared with nonanemic children. In this study, we tested this hypothesis. To measure the anemia level, a HemoCue device was used in both NFHS-3 and NFHS-4 for testing capillary blood samples. A detailed protocol for collecting blood samples and processing them has been elaborated in NFHS's survey manuals. Children with a hemoglobin concentration of <11.0 g/dL were considered anemic (9).

### Statistical approach

A combination of bivariate and multivariate analyses was used. Bivariate analysis was used to assess the proportional difference of children who received WIS, and how it varies with anemia status of children by states/union territories and select background characteristics. Linear regression was run to assess the association between WIS receipt and anemia burden in 640 districts of India. By running a chi-squared (χ^2^) test, the sample of 199,110 was compared with the sample of 8978 excluded from the analysis, to check if both samples differ from each other by age and sex. Comparing both samples, a χ^2^ level of significance of ≥0.05 indicates no difference between the included and excluded samples, thus reducing the probability of sample selection bias. Variance inflation factors (VIFs) were estimated for the potential predictors of receiving WIS to understand the probability of multicollinearity (27), followed by unadjusted logistic regression analysis, and adjusted multivariate logistic regression analysis to examine whether the factors were associated with WIS. A VIF of <5 represented a low probability of multicollinearity (27). The unadjusted logistic regression showed the association between 1 predictor variable and WIS, with the multivariate regression model representing the net association of all predictor variables with WIS. Except counts, all estimates were presented using appropriate sample weighting. The statistical software Stata version 14 (28) was used for all statistical analysis, and OR with 95% CI obtained from logistic regression models are discussed against level of significance (*P*) of <0.05 (2-tailed).

### Ethics statement

NFHS is a publicly available dataset which can be used by researchers upon being granted access by the DHS Program – https://dhsprogram.com/. NFHS was conducted with prior approval from the Institutional Review Board of the nodal agency, appointed by the Ministry of Health and Family Welfare, Government of India. Prior to making NFHS data public, all participant identifiers were removed from the dataset, thus no separate ethical approval was required for this study.

## Results

As 8978 children were excluded from a sample of 208,088 children and left 199,110 children for analyzing factors associated with WIS receipt, a test of sample selection bias was conducted by comparing the age and sex of 8978 children with 199,110 children. A chi-squared (χ^2^) test found that the excluded sample was not statistically different from the sample included in the analysis (*P* ≥0.05), with estimations of χ^2^ = 5.87; *P* = 0.209 for age, and χ^2^ = 2.31; *P* = 0.129 for sex.

The prevalence of anemia among children, the percentage of children who received WIS, and how WIS receipt varies with anemia status in 28 states/union territories were estimated for NFHS-3 (2005–2006) and NFHS-4 (2015–2016), as presented in **Table 1**. Overall, the prevalence of anemia decreased from 69.6% in NFHS-3 to 58.7% in NFHS-4, and WIS receipt also increased from 4.6% in NFHS-3 to 26% in NFHS-4. In the state of Goa and in the union territory of Delhi, although WIS receipt increased over time, the prevalence of anemia also increased. Nationally, though negligible, anemic children had a lower prevalence of receiving WIS compared with nonanemic children, in both rounds of NFHS.

**TABLE 1:** Prevalence of anemia among children, prevalence of children who received weekly iron supplementation (WIS), and prevalence of anemic and nonanemic children who received WIS during 2005–2006, and 2015–2016

	Children with anemia, %		Children who received WIS, %		Anemic children who received WIS, %		Nonanemic children who received WIS, %	
India/states/union territories	2005–2006	2015–2016	2005–2006	2015–2016	2005–2006	2015–2016	2005–2006	2015–2016
India	69.6	58.7	4.6	26.0	4.5	25.6	4.7	26.6
States								
Andhra Pradesh (including Telangana)^1^	70.9	59.4	7.2	31.3	7.1	31.3	7.6	31.2
Arunachal Pradesh	58.2	54.9	4.3	21.0	4.0	19.8	4.6	22.5
Assam	69.2	35.9	0.9	21.2	1.0	20.1	0.5	21.8
Bihar	77.9	63.5	2.8	22.1	2.9	22.1	2.4	22.1
Chhattisgarh	72.2	41.6	3.0	36.3	3.2	34.2	2.6	37.8
Delhi	57.0	60.1	9.2	23.8	9.5	21.7	8.7	26.9
Goa	38.9	48.4	16.8	55.1	16.7	52.9	16.9	57.2
Gujarat	70.0	62.4	10.6	33.4	10.8	33.0	9.9	33.9
Haryana	72.5	71.8	4.3	41.3	4.3	40.2	4.3	44.1
Himachal Pradesh	54.8	53.7	4.5	20.0	2.7	19.6	6.7	20.6
Jammu & Kashmir (including Ladakh)^2^	58.8	54.2	4.5	19.3	3.4	18.3	6.1	20.4
Jharkhand	70.9	70.1	3.	17.5	3.4	17.7	4.8	17.0
Karnataka	70.9	61.2	12.2	50.1	12.9	51.9	10.6	47.3
Kerala	44.6	36.0	6.9	17.9	7.6	16.6	6.3	18.7
Madhya Pradesh	74.1	69.0	3.7	26.2	3.3	26.1	4.9	26.5
Maharashtra	63.3	53.9	7.2	41.1	8.2	41.2	5.6	41.0
Manipur	41.6	24.0	2.2	4.5	2.6	5.0	2.0	4.3
Meghalaya	65.0	48.3	4.3	30.0	4.7	32.7	3.5	27.5
Mizoram	44.5	19.6	22.1	25.0	16.0	15.3	27.0	27.4
Odisha	65.4	44.6	5.3	28.0	4.9	28.2	5.9	27.8
Punjab	66.1	56.7	5.1	32.6	5.3	32.8	4.8	32.2
Rajasthan	70.6	60.5	0.9	14.2	0.6	14.0	1.6	14.6
Sikkim	58.5	55.7	10.5	50.1	8.2	53.1	13.7	46.3
Tamil Nadu	63.5	50.8	9.6	33.9	9.6	34.2	9.6	33.7
Tripura	63.2	47.8	3.3	7.7	3.3	7.4	3.2	8.0
Uttar Pradesh	74.0	63.4	1.3	13.1	1.4	12.8	1.0	13.7
Uttarakhand	61.9	60.1	3.8	14.5	4.1	13.9	3.1	15.5
West Bengal	60.9	54.4	4.6	28.1	4.8	28.2	4.3	27.9

1 During survey period of the 2005–2006 National Family Health Survey, Telangana was part of Andhra Pradesh.

2 During survey period of the 2005–2006 National Family Health Survey, Ladakh was part of Jammu & Kashmir.

**FIGURE 2:**
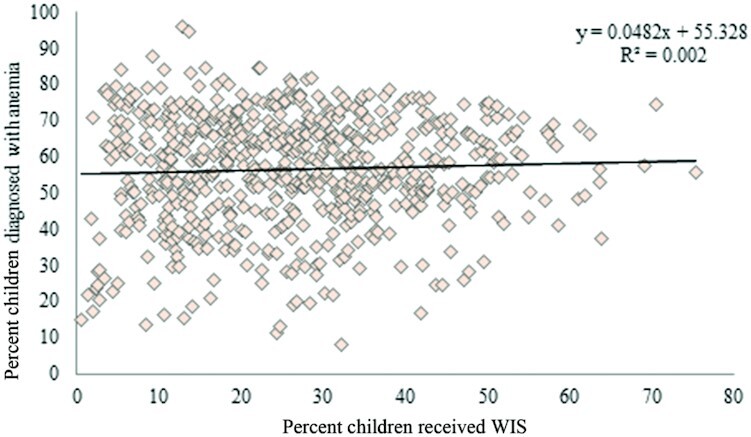
Relation between prevalence (%) of children who received weekly iron supplementation (WIS) and prevalence (%) of anemia among children across 640 districts of India, 2015–2016.

In **Supplementary Table 1**, from NFHS-4, prevalence (%) of children who received WIS and prevalence of children who were diagnosed with anemia in 640 districts was estimated. The lowest coverage of WIS receipt among children was estimated in the Longleng district of Nagaland (0.5%), and the highest was in the Mandya district of Karnataka (75.3%). To understand the district-wise relation between WIS receipt and prevalence of anemia among children, **Figure 2** was developed. With an R^2^ value of 0.002 in linear regression, this relation seems to be very weak.

Prevalence of children receiving WIS by select background characteristics and factors associated with it are presented in **Table 2**. Prior to running the unadjusted and adjusted multivariate logistic regression analysis, VIF was estimated to verify multicollinearity. All VIF values were <5 indicating the probability of multicollinearity is low (data not shown separately). OR with 95% CI estimated from multivariate logistic regression analysis indicates that with increased age, children were more likely to receive WIS, and a similar finding was observed in the unadjusted model. Compared with children of first birth order, children of fifth or higher birth order were less likely to receive WIS (OR: 0.81, CI: 0.75–0.87, *P* <0.001). Children living in rural areas were more likely to receive WIS (OR: 1.05, 95% CI: 1.00–1.11, *P* = 0.042), which is contradictory to the unadjusted analysis. Children from households practicing Islam or Christianity had lower odds of receiving WIS, compared with children from families practicing Hinduism. ST children were more likely to receive WIS (OR: 1.21, CI: 1.13–1.31, *P* <0.001) compared with children belonging to the “others” social group, but no association for this group was observed in the unadjusted model. Children belonging to the richest economic group were less likely to receive WIS (OR: 0.89, CI: 0.82–0.96, *P* = 0.004). Children born to mothers in the age group of 25–29 and children of mothers with education of secondary or higher had higher odds of receiving WIS compared with the referent population. Where mass media exposure was positively associated, the noninstitutional birth of children and children from high-focus group states were negatively associated with WIS receipt. Multivariate analysis showed no association between anemia status and receipt of WIS.

**TABLE 2:** Prevalence of children receiving weekly iron supplementation (WIS), and factors associated with receiving WIS

	Anemic children who received WIS		Unadjusted model		Adjusted model	
	Total sample (unweighted)	% (95% CI)	OR (95% CI)	*P*	OR (95% CI)	*P*
Child age, y						
0	20,998	23.5 (22.9–24.1)	1.00 (referent)		1.00 (referent)	
1	44,356	26.6 (26.2–27.1)	1.18 (1.12–1.25)	<0.001	1.19 (1.12–1.26)	<0.001
2	44,042	26.7 (26.3–27.1)	1.19 (1.12–1.25)	<0.001	1.21 (1.14–1.27)	<0.001
3	45,861	25.9 (25.5–26.3)	1.14 (1.08–1.20)	<0.001	1.16 (1.10–1.23)	<0.001
4	43,853	25.1 (24.7–25.5)	1.09 (1.03–1.15)	0.002	1.13 (1.06–1.19)	<0.001
Sex of child						
Male	103,781	26.0 (25.7–26.3)	1.00 (referent)		1.00 (referent)	
Female	95,329	25.6 (25.3–25.9)	0.98 (0.95–1.01)	0.157	0.98 (0.95–1.01)	0.165
Child's birth order						
First	72,815	27.7 (27.4–28.0)	1.00 (referent)		1.00 (referent)	
Second	61,738	27.3 (27.0–27.7)	0.98 (0.95–1.01)	0.275	1.03 (0.99–1.06)	0.103
Third	32,180	24.2 (23.7–24.7)	0.83 (0.80–0.87)	<0.001	1.01 (0.96–1.05)	0.779
Fourth	16,133	21.5 (20.8–22.2)	0.72 (0.68–0.76)	<0.001	0.98 (0.92–1.04)	0.508
Fifth or higher	16,244	16.7 (16.1–17.4)	0.52 (0.49–0.56)	<0.001	0.81 (0.75–0.87)	<0.001
Area of residence						
Urban	47,559	28.2 (27.8–28.6)	1.00 (referent)		1.00 (referent)	
Rural	151,551	24.9 (24.7–25.1)	0.84 (0.81–0.88)	<0.001	1.05 (1.00–1.11)	0.042
Religion						
Hinduism	148,425	26.1 (25.9–26.4)	1.00 (referent)		1.00 (referent)	
Islam	26,432	22.5 (22.0–23.0)	0.82 (0.78–0.87)	<0.001	0.91 (0.86–0.97)	0.002
Christianity	16,284	27.2 (25.8–28.6)	1.05 (0.94–1.18)	0.361	0.75 (0.66–0.84)	<0.001
Sikhism	3373	33.1 (31.3–34.9)	1.40 (1.26–1.54)	<0.001	0.98 (0.88–1.09)	0.722
Others	4596	31.3 (29.7–33.0)	1.29 (1.09–1.52)	0.003	1.00 (0.85–1.18)	0.984
Social group						
Others	36,171	27.6 (27.1–28.0)	1.00 (referent)		1.00 (referent)	
Scheduled castes	39,562	26.2 (25.8–26.6)	0.93 (0.88–0.99)	0.016	1.00 (0.94–1.06)	0.967
Scheduled tribes	40,990	28.2 (27.5–28.8)	1.03 (0.96–1.10)	0.400	1.21 (1.13–1.31)	<0.001
Other backward classes	82,387	24.3 (24.0–24.6)	0.84 (0.80–0.88)	<0.001	0.96 (0.91–1.01)	0.081
Wealth index						
Poorest	52,863	20.9 (20.6–21.3)	1.00 (referent)		1.00 (referent)	
Poorer	46,344	24.5 (24.1–24.9)	1.23 (1.17–1.29)	<0.001	0.95 (0.90–1.00)	0.043
Middle	39,540	27.6 (27.1–28.0)	1.44 (1.37–1.51)	<0.001	0.93 (0.88–0.99)	0.024
Richer	33,290	29.5 (29.0–29.9)	1.58 (1.50–1.67)	<0.001	0.94 (0.88–1.01)	0.095
Richest	27,073	29.3 (28.8–29.9)	1.57 (1.48–1.66)	<0.001	0.89 (0.82–0.96)	0.004
Mother's age (in years) at birth of her children						
<17	12,179	24.0 (23.3–24.8)	1.00 (referent)		1.00 (referent)	
17–19	58,793	24.8 (24.5–25.2)	1.04 (0.97–1.13)	0.260	1.01 (0.93–1.09)	0.877
20–24	98,072	25.8 (25.5–26.0)	1.10 (1.02–1.18)	0.014	1.03 (0.96–1.11)	0.422
25–29	24,666	29.0 (28.4–29.6)	1.29 (1.19–1.40)	<0.001	1.10 (1.01–1.21)	0.028
≥30	5400	28.3 (27.0–29.7)	1.25 (1.10–1.42)	0.001	1.06 (0.93–1.21)	0.355
Mother's education						
No or incomplete primary	75,422	21.6 (21.3–21.9)	1.00 (referent)		1.00 (referent)	
Primary or incomplete secondary	89,005	27.4 (27.1–27.7)	1.37 (1.31–1.42)	<0.001	1.02 (0.97–1.07)	0.401
Secondary or higher	34,683	30.3 (29.8–30.7)	1.57 (1.50–1.65)	<0.001	1.10 (1.03–1.17)	0.003
Delivery care						
Institutional	149,862	27.8 (27.6–28.0)	1.00 (referent)		1.00 (referent)	
Noninstitutional	49,248	18.4 (18.0–18.7)	0.58 (0.56–0.61)	<0.001	0.73 (0.70–0.77)	<0.001
Mass media exposure of mother						
No exposure	55,297	19.5 (19.1–19.8)	1.00 (referent)		1.00 (referent)	
Some exposure	143,813	28.1 (27.9–28.4)	1.62 (1.56–1.68)	<0.001	1.18 (1.12–1.24)	<0.001
States/UT						
Nonhigh focus group states/UT	75,680	34.1 (33.8–34.5)	1.00 (referent)		1.00 (referent)	
High focus group states/UT	123,430	19.4 (19.2–19.7)	0.47 (0.45–0.48)	<0.001	0.51 (0.49–0.53)	<0.001
Anemia status of children						
Anemic	115,019	25.4 (25.2–25.7)	1.00 (referent)		1.00 (referent)	
Nonanemic	84,091	26.4 (26.1–26.7)	1.05 (1.02–1.08)	0.002	1.00 (0.97–1.04)	0.918
Total	199,110	25.8 (25.6–26.0)				

UT, union territories.

*P* represents the level of significance estimated from the logistic regression model; (referent) indicates the reference category assigned in the logistic regression model.

## Discussion

To reduce an unacceptably high burden of anemia, the Indian government introduced NIPI in 2011, which prescribes the biweekly IFA supplementation for children aged 6–59 mo. Data to analyze the performance of the NIPI program are not available. Instead, using 2 consecutive waves of nationally representative NFHS data, this study examined the trend of receiving WIS among children aged 6–59 mo between 2005–2006 and 2015–2016, and factors associated with receiving WIS in 2015–2016.

Findings reveal that with increased age, children were more likely to receive WIS. This finding is consistent with studies conducted in Africa, which showed that with increasing age, children aged 6–59 mo were more likely to receive healthcare (29), as is true in India (30). Although the association of WIS with increased age demands further investigation, one can safely comment that compliance with WIS among children aged under 1 y is lower than among relatively older children. Previous studies conducted in India (31) and Nigeria (32) demonstrated that children from higher birth order had lower odds of healthcare utilization, which may be reflected in this study's findings that children from fifth or higher birth order had a lower chance of receiving WIS. Compared with children living in urban areas, children from rural areas were more likely to receive WIS. This finding explains the government's focused intervention in rural India as children of rural India have a higher chance of being anemic (14). However, underserved children in urban areas (notably in urban slums of cities like Mumbai, Delhi, Kolkata, or Chennai) deserve greater attention for WIS. The effect of religion on healthcare utilization in India is complex, and why children from families practicing a certain religion did not receive appropriate healthcare is difficult to adequately explain. Findings of this study indicate that compared with children from Hindu households, children from families following Islam or Christianity had lower odds of receiving WIS. Although this finding warrants further investigation, a previous study also found that followers of Islam had lower odds of healthcare utilization (33).

Children from ST households had higher odds of receiving WIS compared with children belonging to the “Others” social group. Among all social groups, STs are considered the most disadvantaged with lower income and education, whereas “others” are historically a more privileged group (34); also, STs generally have low healthcare service utilization for their children (35) and higher WIS receipt could be attributed to success of the government of targeting the NIPI program to ST children. Receipt of WIS among the richest children is lower than among the poorest children. As with the case of higher levels of WIS consumption in rural areas which are heavily targeted by the government, higher income areas may be neglected under programs like NIPI, as it is assumed that children in these households are less deprived. However, this hypothesis would require further investigation. Children born to mothers between the age of 25 and 29 y, and children of mothers with secondary education or higher were positively associated with receiving WIS. Mother's education and mother's age at childbirth could be linked (36) as the birth of children between the age of 25 and 29 y is indicative of higher age at marriage and opportunity of education of the mother which may lead to better healthcare utilization for their children (37). Exposure to mass media and children's birth at a healthcare facility could increase the odds of receiving WIS, as both events have the potential to educate mothers about the importance of specific child healthcare practices, and in this case, mothers seemed to have had some exposure to the importance of WIS for their children. Finally, although some improvement in receipt of WIS was observed during 2005–2016, multivariate analysis reveals that receipt of WIS was not associated with anemia status of children. This finding may be explained by the fact that WIS intervention, either by public or private institution, is not contingent upon a child's anemia status, and is given to all children aged 6–59 mo. Secondly, this finding could be indicative of the fact that the strategy of testing and treating anemia was not put in place effectively. This finding does not suggest that the WIS intervention is not an effective intervention to reduce the burden of anemia and careful interpretation of this finding is needed.

The findings of this study are not free from caveats. First, results should be interpreted with caution as this study uses cross-sectional data, thus causal inference should be avoided. Second, most of the information about children is based on the recall of mothers. Any errors in recall are likely to be nondifferential with respect to the predictors being examined and thus the associations may have been stronger. Third, all potential predictors of receipt of WIS were not available with the dataset used (for example, adequacy of supply of iron tablet or syrup). Despite these limitations, the analysis of nationally representative data with high external validity is the primary strength of the study, and these findings could be useful for strengthening the ongoing NIPI program as well as anemia reduction policy for children.

To conclude, this study finds that WIS receipt has increased between 2005–2006 and 2015–2016, but the prevalence of WIS still remains unacceptably low (in absolute terms), and the gap in district-wise WIS coverage is substantial. Although there is some indication of success of the NIPI program in pockets, a proper evaluation of NIPI is needed. WIS to children was not associated with their anemia status, therefore the NIPI program should focus greater attention on districts with higher anemia burden. Furthermore, children of fifth or higher birth order, non-Hindu household children, higher income household children, children not born at a health facility, and children from high-focus group states should receive special attention as they had low access to WIS.

## Supplementary Material

nzab020_Supplemental_File

## Data Availability

NFHS data used for this study can be accessed from the official website of the DHS Program - https://dhsprogram.com/.

## Notes

This work was supported by the West Bengal State Department of Health and Family Welfare, India (Memo number: 114-P&B/ HFW-27011/114/2019-NHM SEC). The funders had no role in the design/conduct of the study, collection/analysis/interpretation of the data, and preparation/review/approval of the manuscript.

Author disclosures: The authors report no conflicts of interest.

Supplemental Table 1 is available from the “Supplementary data” link in the online posting of the article and from the same link in the online table of contents at https://academic.oup.com/cdn/.

Abbreviations used: DHS, Demographic and Health Survey; IDA, iron deficiency anemia; IFA, iron-and-folic-acid; NFHS, National Family Health Survey; NIPI, National Iron Plus Initiative; ST, Scheduled Tribe; VIF, variance inflation factor; WIS, weekly iron supplementation.
