# Supplementary material for: Receipt of Weekly Iron Supplementation among Indian Children, 2005–2016
Source: Curr Dev Nutr. 2021 Mar 3;5(3):nzab020. doi: 10.1093/cdn/nzab020 (PMC8164210; doi:10.1093/cdn/nzab020)
Supplement: nzab020_Supplemental_File [file nzab020_supplemental_file.docx]

Receipt of Weekly Iron Supplementation among Indian Children, 2005-2016

Rajesh Kumar Rai

**Online Supplementary Material**

| **Supplementary Table 1**. Prevalence (%) of children who received weekly iron supplementation (WIS) and prevalence of children who were diagnosed with anaemia in 640 districts of India, 2015-2016 | | | |
| --- | --- | --- | --- |
| States / union territories | Districts | Received WIS, % | Diagnosed with anaemia, % |
| Andaman and Nicobar Islands | North and Middle Andaman | 17.4 | 46.8 |
| Andaman and Nicobar Islands | Nicobar | 25.6 | 53.8 |
| Andaman and Nicobar Islands | South Andaman | 27.7 | 51.5 |
|  |  |  |  |
| Andhra Pradesh | Kurnool | 16.5 | 54.7 |
| Andhra Pradesh | Krishna | 20.4 | 57.4 |
| Andhra Pradesh | East Godavari | 21.8 | 64.6 |
| Andhra Pradesh | Visakhapatnam | 23.1 | 63.2 |
| Andhra Pradesh | Vizianagaram | 26.0 | 78.2 |
| Andhra Pradesh | Sri Potti Sriramulu | 26.1 | 50.1 |
| Andhra Pradesh | Guntur | 26.7 | 66.4 |
| Andhra Pradesh | Anantapur | 26.7 | 53.1 |
| Andhra Pradesh | West Godavari | 31.3 | 52.9 |
| Andhra Pradesh | Chittoor | 33.8 | 48.0 |
| Andhra Pradesh | Kadapa | 34.7 | 56.2 |
| Andhra Pradesh | Prakasam | 36.5 | 56.3 |
| Andhra Pradesh | Srikakulam | 45.6 | 70.1 |
|  |  |  |  |
| Arunachal Pradesh | Changlang | 3.5 | 63.2 |
| Arunachal Pradesh | Tirap | 4.2 | 59.2 |
| Arunachal Pradesh | Tawang | 9.0 | 69.8 |
| Arunachal Pradesh | East Kameng | 10.0 | 49.3 |
| Arunachal Pradesh | Kurung Kumey | 13.0 | 50.5 |
| Arunachal Pradesh | Dibang Valley | 13.8 | 46.1 |
| Arunachal Pradesh | West kameng | 17.5 | 51.7 |
| Arunachal Pradesh | Lohit | 18.3 | 57.5 |
| Arunachal Pradesh | West Siang | 24.9 | 40.4 |
| Arunachal Pradesh | Lower Subansiri | 27.2 | 52.3 |
| Arunachal Pradesh | Lower Dibang Valley | 28.3 | 59.8 |
| Arunachal Pradesh | Upper Subansiri | 36.1 | 52.9 |
| Arunachal Pradesh | Upper Siang | 37.4 | 51.5 |
| Arunachal Pradesh | Papumpare | 38.7 | 55.4 |
| Arunachal Pradesh | Anjaw | 40.3 | 62.4 |
| Arunachal Pradesh | East Siang | 42.0 | 46.4 |
|  |  |  |  |
| Assam | Karbi Anglong | 9.4 | 25.1 |
| Assam | Morigaon | 9.4 | 38.4 |
| Assam | Nagaon | 10.9 | 35.9 |
| Assam | Lakhimpur | 12.3 | 32.4 |
| Assam | Jorhat | 12.3 | 37.6 |
| Assam | Dhemaji | 12.5 | 39.0 |
| Assam | Dima hasao | 12.5 | 29.5 |
| Assam | Kokrajhar | 13.6 | 40.5 |
| Assam | Dhubri | 13.8 | 43.5 |
| Assam | Dibrugarh | 15.1 | 52.4 |
| Assam | Kamrup Metropolitan | 18.2 | 34.1 |
| Assam | Tinsukia | 18.8 | 43.9 |
| Assam | Kamrup | 19.2 | 34.3 |
| Assam | Nalbari | 20.4 | 46.2 |
| Assam | Barpeta | 20.8 | 30.2 |
| Assam | Bongaigaon | 21.6 | 34.7 |
| Assam | Darrang | 21.8 | 45.2 |
| Assam | Sivasagar | 23.3 | 34.3 |
| Assam | Baksa | 24.2 | 39.4 |
| Assam | Cachar | 26.8 | 30.0 |
| Assam | Golaghat | 27.4 | 33.4 |
| Assam | Sonitpur | 29.3 | 27.1 |
| Assam | Chirang | 29.3 | 35.1 |
| Assam | Goalpara | 30.7 | 35.6 |
| Assam | Udalguri | 36.7 | 39.2 |
| Assam | Karimganj | 44.6 | 24.5 |
| Assam | Hailakandi | 47.8 | 28.0 |
|  |  |  |  |
| Bihar | Kaimur (Bhabua) | 8.9 | 63.5 |
| Bihar | Aurangabad | 10.4 | 52.9 |
| Bihar | Rohtas | 11.4 | 61.4 |
| Bihar | Arwal | 11.8 | 67.1 |
| Bihar | Begusarai | 11.9 | 63.3 |
| Bihar | Madhepura | 13.0 | 61.5 |
| Bihar | Purba Champaran | 13.8 | 65.7 |
| Bihar | Muzaffarpur | 14.7 | 59.2 |
| Bihar | Gaya | 14.9 | 58.8 |
| Bihar | Nalanda | 15.1 | 58.8 |
| Bihar | Katihar | 15.4 | 61.9 |
| Bihar | Jamui | 17.2 | 61.1 |
| Bihar | Bhojpur | 17.7 | 71.2 |
| Bihar | Sheohar | 17.8 | 63.2 |
| Bihar | Lakhisarai | 18.0 | 67.1 |
| Bihar | Banka | 19.3 | 70.0 |
| Bihar | Buxar | 20.1 | 59.8 |
| Bihar | Gopalganj | 21.6 | 63.8 |
| Bihar | Patna | 22.2 | 51.6 |
| Bihar | Khagaria | 22.3 | 63.4 |
| Bihar | Madhubani | 22.5 | 63.9 |
| Bihar | Saharsa | 23.6 | 67.7 |
| Bihar | Nawada | 25.0 | 55.8 |
| Bihar | Sitamarhi | 25.2 | 68.2 |
| Bihar | Saran | 25.3 | 61.6 |
| Bihar | Sheikhpura | 26.9 | 66.1 |
| Bihar | Munger | 26.9 | 63.0 |
| Bihar | Jehanabad | 27.6 | 62.4 |
| Bihar | Siwan | 28.0 | 63.1 |
| Bihar | Darbhanga | 28.5 | 69.2 |
| Bihar | Vaishali | 29.0 | 66.9 |
| Bihar | Bhagalpur | 29.7 | 70.2 |
| Bihar | Araria | 30.0 | 61.3 |
| Bihar | Kishanganj | 30.8 | 65.5 |
| Bihar | Supaul | 31.0 | 72.7 |
| Bihar | Purnia | 31.0 | 67.3 |
| Bihar | Samastipur | 31.1 | 65.2 |
| Bihar | Pashchim Champaran | 31.3 | 62.3 |
|  |  |  |  |
| Chandigarh | Chandigarh | 13.8 | 72.2 |
|  |  |  |  |
| Chhattisgarh | Janjgir - Champa | 19.4 | 35.5 |
| Chhattisgarh | Mahasamund | 26.1 | 37.4 |
| Chhattisgarh | Dakshin Bastar Dantewada | 28.3 | 71.1 |
| Chhattisgarh | Korba | 31.2 | 38.9 |
| Chhattisgarh | Durg | 31.6 | 44.9 |
| Chhattisgarh | Jashpur | 32.0 | 31.3 |
| Chhattisgarh | Bastar | 32.6 | 58.8 |
| Chhattisgarh | Uttar Bastar Kanker | 33.8 | 61.4 |
| Chhattisgarh | Dhamtari | 34.3 | 53.3 |
| Chhattisgarh | Surguja | 35.8 | 38.3 |
| Chhattisgarh | Kabirdham | 36.3 | 37.5 |
| Chhattisgarh | Raigarh | 36.5 | 39.5 |
| Chhattisgarh | Raipur | 38.8 | 46.7 |
| Chhattisgarh | Narayanpur | 41.3 | 48.0 |
| Chhattisgarh | Rajnandgaon | 42.2 | 30.0 |
| Chhattisgarh | Bijapur | 42.7 | 50.8 |
| Chhattisgarh | Korea (Koriya) | 45.6 | 33.6 |
| Chhattisgarh | Bilaspur | 49.6 | 31.1 |
|  |  |  |  |
| Dadra and Nagar Haveli | Dadra and Nagar Haveli | 15.7 | 84.4 |
|  |  |  |  |
| Daman and Diu | Daman | 23.5 | 74.6 |
| Daman and Diu | Diu | 37.1 | 72.5 |
|  |  |  |  |
| Goa | South Goa | 46.9 | 48.2 |
| Goa | North Goa | 61.1 | 48.5 |
|  |  |  |  |
| Gujarat | Gandhinagar | 14.7 | 74.2 |
| Gujarat | Kheda | 24.0 | 55.2 |
| Gujarat | The Dangs | 24.3 | 74.1 |
| Gujarat | Banaskantha | 24.8 | 56.3 |
| Gujarat | Bharuch | 25.3 | 56.5 |
| Gujarat | Vadodara | 25.4 | 55.2 |
| Gujarat | Jamnagar | 26.4 | 76.3 |
| Gujarat | Anand | 27.1 | 57.8 |
| Gujarat | Navsari | 27.7 | 51.6 |
| Gujarat | Kachchh | 28.1 | 81.1 |
| Gujarat | Narmada | 28.8 | 53.4 |
| Gujarat | Ahmadabad | 29.5 | 76.9 |
| Gujarat | Rajkot | 30.5 | 56.3 |
| Gujarat | Panchmahal | 31.6 | 50.6 |
| Gujarat | Sabarkantha | 34.6 | 72.6 |
| Gujarat | Surendranagar | 34.7 | 75.6 |
| Gujarat | Tapi | 35.3 | 49.3 |
| Gujarat | Porbandar | 35.4 | 70.5 |
| Gujarat | Mahesana | 35.7 | 77.0 |
| Gujarat | Amreli | 39.2 | 74.0 |
| Gujarat | Dohad | 39.3 | 58.4 |
| Gujarat | Junagadh | 43.0 | 76.2 |
| Gujarat | Valsad | 44.7 | 49.5 |
| Gujarat | Surat | 45.4 | 40.9 |
| Gujarat | Patan | 48.0 | 66.3 |
| Gujarat | Bhavnagar | 49.9 | 70.4 |
|  |  |  |  |
| Haryana | Mewat | 10.6 | 84.2 |
| Haryana | Sirsa | 32.1 | 72.4 |
| Haryana | Palwal | 32.5 | 76.1 |
| Haryana | Bhiwani | 33.1 | 74.5 |
| Haryana | Rewari | 38.1 | 78.6 |
| Haryana | Jind | 41.0 | 76.4 |
| Haryana | Rohtak | 41.8 | 76.4 |
| Haryana | Karnal | 42.1 | 75.5 |
| Haryana | Kaithal | 42.6 | 67.5 |
| Haryana | Yamunanagar | 43.3 | 57.7 |
| Haryana | Kurukshetra | 44.4 | 63.0 |
| Haryana | Fatehabad | 44.7 | 70.3 |
| Haryana | Faridabad | 47.2 | 74.6 |
| Haryana | Gurgaon | 47.5 | 66.5 |
| Haryana | Panchkula | 47.6 | 66.2 |
| Haryana | Panipat | 48.3 | 65.3 |
| Haryana | Ambala | 50.2 | 75.0 |
| Haryana | Mahendragarh | 51.2 | 73.8 |
| Haryana | Sonipat | 51.5 | 59.5 |
| Haryana | Hisar | 53.3 | 65.8 |
| Haryana | Jhajjar | 54.4 | 70.9 |
|  |  |  |  |
| Himachal Pradesh | Chamba | 6.0 | 65.5 |
| Himachal Pradesh | Lahul and Spiti | 13.6 | 94.8 |
| Himachal Pradesh | Mandi | 13.6 | 37.2 |
| Himachal Pradesh | Kinnaur | 13.8 | 83.2 |
| Himachal Pradesh | Shimla | 15.0 | 70.3 |
| Himachal Pradesh | Kullu | 15.8 | 54.7 |
| Himachal Pradesh | Sirmaur | 20.1 | 65.8 |
| Himachal Pradesh | Kangra | 24.4 | 47.8 |
| Himachal Pradesh | Solan | 24.7 | 71.5 |
| Himachal Pradesh | Una | 27.5 | 54.5 |
| Himachal Pradesh | Bilaspur | 29.3 | 29.9 |
| Himachal Pradesh | Hamirpur | 32.8 | 40.8 |
|  |  |  |  |
| Jammu and Kashmir | Samba | 7.3 | 46.0 |
| Jammu and Kashmir | Bandipore | 10.7 | 57.9 |
| Jammu and Kashmir | Shupiyan | 10.7 | 60.3 |
| Jammu and Kashmir | Pulwama | 10.9 | 53.5 |
| Jammu and Kashmir | Baramula | 12.7 | 62.2 |
| Jammu and Kashmir | Kupwara | 12.7 | 74.8 |
| Jammu and Kashmir | Kulgam | 14.1 | 59.1 |
| Jammu and Kashmir | Kathua | 14.7 | 41.5 |
| Jammu and Kashmir | Jammu | 14.8 | 34.6 |
| Jammu and Kashmir | Badgam | 19.3 | 71.0 |
| Jammu and Kashmir | Anantnag | 20.2 | 39.7 |
| Jammu and Kashmir | Reasi | 21.2 | 63.3 |
| Jammu and Kashmir | Srinagar | 21.4 | 51.9 |
| Jammu and Kashmir | Ganderbal | 21.6 | 51.6 |
| Jammu and Kashmir | Doda | 21.8 | 56.6 |
| Jammu and Kashmir | Udhampur | 26.1 | 64.8 |
| Jammu and Kashmir | Punch | 26.2 | 51.5 |
| Jammu and Kashmir | Rajouri | 27.5 | 51.5 |
| Jammu and Kashmir | Kishtwar | 34.3 | 64.3 |
| Jammu and Kashmir | Ramban | 41.1 | 50.2 |
|  |  |  |  |
| Jharkhand | Sahibganj | 2.0 | 70.7 |
| Jharkhand | Pashchimi Singhbhum | 5.4 | 83.9 |
| Jharkhand | Dhanbad | 5.4 | 69.7 |
| Jharkhand | Garhwa | 8.5 | 65.5 |
| Jharkhand | Bokaro | 11.3 | 73.8 |
| Jharkhand | Palamu | 11.4 | 59.3 |
| Jharkhand | Dumka | 11.5 | 75.1 |
| Jharkhand | Jamtara | 11.9 | 73.6 |
| Jharkhand | Purbi Singhbhum | 14.0 | 67.5 |
| Jharkhand | Ramgarh | 14.4 | 71.8 |
| Jharkhand | Latehar | 15.6 | 50.8 |
| Jharkhand | Godda | 17.9 | 81.3 |
| Jharkhand | Simdega | 18.6 | 79.8 |
| Jharkhand | Chatra | 19.1 | 60.5 |
| Jharkhand | Kodarma | 19.5 | 72.2 |
| Jharkhand | Pakur | 20.8 | 74.3 |
| Jharkhand | Giridih | 22.7 | 74.7 |
| Jharkhand | Deoghar | 25.2 | 64.8 |
| Jharkhand | Gumla | 28.1 | 73.5 |
| Jharkhand | Saraikela Kharsawan | 28.7 | 81.8 |
| Jharkhand | Ranchi | 29.3 | 65.8 |
| Jharkhand | Khunti | 30.6 | 64.5 |
| Jharkhand | Hazaribagh | 32.0 | 64.8 |
| Jharkhand | Lohardaga | 35.6 | 65.6 |
|  |  |  |  |
| Karnataka | Bangalore | 37.8 | 52.7 |
| Karnataka | Gulbarga | 42.2 | 72.4 |
| Karnataka | Bijapur | 42.4 | 68.1 |
| Karnataka | Dakshina Kannada | 42.5 | 54.2 |
| Karnataka | Mysore | 43.7 | 59.4 |
| Karnataka | Bidar | 43.7 | 69.4 |
| Karnataka | Uttara Kannada | 45.2 | 49.0 |
| Karnataka | Davanagere | 45.5 | 66.5 |
| Karnataka | Kodagu | 47.8 | 46.5 |
| Karnataka | Chitradurga | 49.4 | 64.6 |
| Karnataka | Bagalkot | 49.9 | 63.3 |
| Karnataka | Tumkur | 49.9 | 54.4 |
| Karnataka | Yadgir | 49.9 | 74.0 |
| Karnataka | Bellary | 50.0 | 73.2 |
| Karnataka | Udupi | 50.4 | 54.3 |
| Karnataka | Shimoga | 51.4 | 54.2 |
| Karnataka | Raichur | 52.6 | 71.5 |
| Karnataka | Hassan | 53.1 | 55.4 |
| Karnataka | Chikmagalur | 53.6 | 61.0 |
| Karnataka | Koppal | 53.6 | 67.3 |
| Karnataka | Dharwad | 55.2 | 50.2 |
| Karnataka | Chikkaballapura | 58.0 | 64.1 |
| Karnataka | Haveri | 58.5 | 63.1 |
| Karnataka | Gadag | 61.2 | 68.7 |
| Karnataka | Bangalore rural | 61.9 | 49.2 |
| Karnataka | Belgaum | 62.4 | 66.0 |
| Karnataka | Ramanagara | 63.7 | 56.4 |
| Karnataka | Chamarajanagar | 63.7 | 52.8 |
| Karnataka | Kolar | 69.1 | 57.3 |
| Karnataka | Mandya | 75.3 | 55.6 |
|  |  |  |  |
| Kerala | Kannur | 7.5 | 43.5 |
| Kerala | Kasaragod | 10.9 | 38.4 |
| Kerala | Malappuram | 13.9 | 54.5 |
| Kerala | Pathanamthitta | 14.1 | 18.6 |
| Kerala | Kozhikode | 15.3 | 40.1 |
| Kerala | Thrissur | 15.6 | 38.1 |
| Kerala | Kottayam | 15.8 | 34.5 |
| Kerala | Thiruvananthapuram | 16.4 | 21.1 |
| Kerala | Alappuzha | 16.7 | 25.7 |
| Kerala | Wayanad | 18.7 | 44.3 |
| Kerala | Palakkad | 18.7 | 41.0 |
| Kerala | Ernakulam | 22.6 | 24.9 |
| Kerala | Idukki | 24.3 | 33.1 |
| Kerala | Kollam | 41.9 | 16.8 |
|  |  |  |  |
| Ladakh | Kargil | 9.2 | 87.5 |
| Ladakh | Leh | 12.8 | 95.8 |
|  |  |  |  |
| Lakshadweep | Lakshadweep | 11.1 | 52.8 |
|  |  |  |  |
| Madhya Pradesh | Guna | 12.9 | 67.5 |
| Madhya Pradesh | Sagar | 13.8 | 67.2 |
| Madhya Pradesh | Morena | 15.3 | 67.3 |
| Madhya Pradesh | Raisen | 15.7 | 67.7 |
| Madhya Pradesh | Shahdol | 16.7 | 65.7 |
| Madhya Pradesh | Vidisha | 16.7 | 70.4 |
| Madhya Pradesh | Bhind | 17.0 | 72.1 |
| Madhya Pradesh | Gwalior | 17.9 | 69.2 |
| Madhya Pradesh | Balaghat | 18.2 | 70.5 |
| Madhya Pradesh | Shivpuri | 18.7 | 63.0 |
| Madhya Pradesh | Datia | 19.6 | 72.7 |
| Madhya Pradesh | Sidhi | 19.9 | 67.6 |
| Madhya Pradesh | Sheopur | 20.1 | 77.2 |
| Madhya Pradesh | Seoni | 20.7 | 61.3 |
| Madhya Pradesh | Panna | 20.9 | 68.7 |
| Madhya Pradesh | Singrauli | 21.5 | 61.5 |
| Madhya Pradesh | Rajgarh | 21.5 | 62.4 |
| Madhya Pradesh | Mandla | 23.3 | 70.0 |
| Madhya Pradesh | Shajapur | 23.9 | 76.8 |
| Madhya Pradesh | Tikamgarh | 24.0 | 68.6 |
| Madhya Pradesh | Khandwa (East Nimar) | 24.4 | 77.2 |
| Madhya Pradesh | Dindori | 24.7 | 67.0 |
| Madhya Pradesh | Barwani | 25.9 | 81.2 |
| Madhya Pradesh | Mandsaur | 25.9 | 65.5 |
| Madhya Pradesh | Burhanpur | 26.1 | 80.4 |
| Madhya Pradesh | Khargone (West Nimar) | 26.1 | 76.8 |
| Madhya Pradesh | Betul | 26.7 | 61.4 |
| Madhya Pradesh | Damoh | 26.9 | 76.7 |
| Madhya Pradesh | Bhopal | 27.1 | 77.1 |
| Madhya Pradesh | Rewa | 27.5 | 54.6 |
| Madhya Pradesh | Chhatarpur | 27.7 | 66.3 |
| Madhya Pradesh | Narsimhapur | 28.2 | 69.5 |
| Madhya Pradesh | Satna | 28.6 | 70.7 |
| Madhya Pradesh | Jabalpur | 28.8 | 59.5 |
| Madhya Pradesh | Alirajpur | 30.3 | 74.5 |
| Madhya Pradesh | Dewas | 31.4 | 65.6 |
| Madhya Pradesh | Indore | 31.8 | 70.8 |
| Madhya Pradesh | Ashoknagar | 32.3 | 59.8 |
| Madhya Pradesh | Anuppur | 33.0 | 67.3 |
| Madhya Pradesh | Katni | 33.6 | 65.4 |
| Madhya Pradesh | Jhabua | 33.8 | 73.5 |
| Madhya Pradesh | Chhindwara | 33.9 | 65.7 |
| Madhya Pradesh | Sehore | 34.2 | 65.4 |
| Madhya Pradesh | Umaria | 34.7 | 72.7 |
| Madhya Pradesh | Ratlam | 34.8 | 76.8 |
| Madhya Pradesh | Hoshangabad | 38.2 | 67.8 |
| Madhya Pradesh | Neemuch | 38.7 | 70.6 |
| Madhya Pradesh | Dhar | 40.0 | 75.8 |
| Madhya Pradesh | Harda | 40.9 | 66.0 |
| Madhya Pradesh | Ujjain | 44.2 | 69.3 |
|  |  |  |  |
| Maharashtra | Nagpur | 18.6 | 44.5 |
| Maharashtra | Akola | 21.9 | 52.9 |
| Maharashtra | Satara | 22.9 | 57.6 |
| Maharashtra | Pune | 29.2 | 54.0 |
| Maharashtra | Latur | 29.4 | 54.3 |
| Maharashtra | Nanded | 29.7 | 52.7 |
| Maharashtra | Nashik | 33.4 | 52.5 |
| Maharashtra | Nandurbar | 33.9 | 60.2 |
| Maharashtra | Osmanabad | 34.2 | 36.5 |
| Maharashtra | Sangli | 34.4 | 49.1 |
| Maharashtra | Jalgaon | 34.9 | 59.8 |
| Maharashtra | Solapur | 35.6 | 51.4 |
| Maharashtra | Yavatmal | 37.9 | 69.2 |
| Maharashtra | Gondiya | 39.6 | 55.9 |
| Maharashtra | Wardha | 40.0 | 46.9 |
| Maharashtra | Gadchiroli | 40.3 | 58.4 |
| Maharashtra | Raigarh | 40.6 | 52.0 |
| Maharashtra | Kolhapur | 40.9 | 44.9 |
| Maharashtra | Ratnagiri | 40.9 | 51.8 |
| Maharashtra | Thane | 41.4 | 55.2 |
| Maharashtra | Jalna | 42.1 | 49.2 |
| Maharashtra | Washim | 42.5 | 60.9 |
| Maharashtra | Hingoli | 44.8 | 50.8 |
| Maharashtra | Amravati | 47.9 | 50.6 |
| Maharashtra | Parbhani | 49.2 | 52.2 |
| Maharashtra | Bid | 50.7 | 58.2 |
| Maharashtra | Chandrapur | 51.1 | 59.2 |
| Maharashtra | Buldana | 51.4 | 43.4 |
| Maharashtra | Dhule | 53.0 | 66.8 |
| Maharashtra | Bhandara | 55.0 | 43.2 |
| Maharashtra | Ahmadnagar | 57.0 | 47.8 |
| Maharashtra | Mumbai | 57.4 | 66.3 |
| Maharashtra | Mumbai Suburban | 58.1 | 68.9 |
| Maharashtra | Sindhudurg | 58.7 | 40.9 |
| Maharashtra | Aurangabad | 63.9 | 37.5 |
|  |  |  |  |
| Manipur | Bishnupur | 1.4 | 21.9 |
| Manipur | Churachandpur | 1.9 | 17.3 |
| Manipur | Tamenglong | 2.4 | 23.5 |
| Manipur | Thoubal | 2.5 | 24.7 |
| Manipur | Chandel | 2.6 | 28.4 |
| Manipur | Imphal East | 2.8 | 20.5 |
| Manipur | Ukhrul | 3.3 | 26.3 |
| Manipur | Senapati | 4.4 | 22.9 |
| Manipur | Imphal West | 11.7 | 29.7 |
|  |  |  |  |
| Meghalaya | West Khasi Hills | 14.7 | 36.1 |
| Meghalaya | Jaintia Hills | 23.3 | 33.4 |
| Meghalaya | Ribhoi | 23.7 | 44.4 |
| Meghalaya | West Garo Hills | 28.9 | 52.8 |
| Meghalaya | East Khasi Hills | 36.3 | 46.4 |
| Meghalaya | East Garo Hills | 39.8 | 75.4 |
| Meghalaya | South Garo Hills | 70.5 | 74.6 |
|  |  |  |  |
| Mizoram | Lawngtlai | 12.1 | 34.2 |
| Mizoram | Aizawl | 24.3 | 11.1 |
| Mizoram | Lunglei | 26.1 | 27.7 |
| Mizoram | Mamit | 26.8 | 19.8 |
| Mizoram | Kolasib | 27.5 | 41.2 |
| Mizoram | Serchhip | 27.7 | 29.6 |
| Mizoram | Saiha | 31.3 | 21.9 |
| Mizoram | Champhai | 32.2 | 7.9 |
|  |  |  |  |
| Nagaland | Longleng | 0.5 | 14.9 |
| Nagaland | Mon | 1.8 | 42.9 |
| Nagaland | Tuensang | 2.8 | 28.6 |
| Nagaland | Kiphire | 5.0 | 25.0 |
| Nagaland | Phek | 8.5 | 13.4 |
| Nagaland | Wokha | 8.7 | 32.2 |
| Nagaland | Zunheboto | 10.7 | 40.4 |
| Nagaland | Peren | 10.7 | 16.4 |
| Nagaland | Kohima | 11.6 | 36.8 |
| Nagaland | Dimapur | 13.0 | 15.4 |
| Nagaland | Mokokchung | 24.8 | 13.0 |
|  |  |  |  |
| Delhi | North East | 18.5 | 47.1 |
| Delhi | South | 20.0 | 67.2 |
| Delhi | West | 22.8 | 65.3 |
| Delhi | North West | 24.0 | 74.4 |
| Delhi | North | 25.8 | 62.2 |
| Delhi | South West | 26.6 | 52.0 |
| Delhi | East | 29.4 | 50.6 |
| Delhi | New Delhi | 29.7 | 66.3 |
| Delhi | Central | 37.4 | 76.7 |
|  |  |  |  |
| Odisha | Nuapada | 13.5 | 64.6 |
| Odisha | Anugul | 17.4 | 38.0 |
| Odisha | Ganjam | 18.3 | 37.2 |
| Odisha | Balangir | 18.8 | 67.3 |
| Odisha | Dhenkanal | 20.6 | 39.1 |
| Odisha | Koraput | 21.5 | 71.6 |
| Odisha | Sambalpur | 21.5 | 69.9 |
| Odisha | Gajapati | 21.6 | 57.5 |
| Odisha | Kendrapara | 22.6 | 28.8 |
| Odisha | Baudh | 23.1 | 44.2 |
| Odisha | Kalahandi | 23.6 | 67.6 |
| Odisha | Jharsuguda | 24.1 | 66.8 |
| Odisha | Baleshwar | 25.6 | 28.0 |
| Odisha | Khordha | 26.3 | 18.9 |
| Odisha | Bargarh | 26.8 | 68.3 |
| Odisha | Sundargarh | 27.1 | 75.4 |
| Odisha | Cuttack | 28.5 | 19.5 |
| Odisha | Debagarh | 29.7 | 30.3 |
| Odisha | Bhadrak | 30.3 | 22.4 |
| Odisha | Nabarangapur | 30.7 | 72.5 |
| Odisha | Kandhamal | 31.4 | 43.3 |
| Odisha | Kendujhar | 32.6 | 32.3 |
| Odisha | Mayurbhanj | 33.0 | 34.0 |
| Odisha | Puri | 34.4 | 29.1 |
| Odisha | Jajapur | 39.5 | 29.6 |
| Odisha | Rayagada | 42.2 | 50.1 |
| Odisha | Malkangiri | 42.8 | 71.7 |
| Odisha | Jagatsinghapur | 43.7 | 24.7 |
| Odisha | Nayagarh | 47.1 | 26.0 |
| Odisha | Subarnapur | 49.8 | 74.5 |
|  |  |  |  |
| Puducherry | Karaikal | 32.2 | 48.2 |
| Puducherry | Yanam | 38.1 | 38.8 |
| Puducherry | Puducherry | 47.9 | 44.6 |
| Puducherry | Mahe | 51.9 | 41.4 |
|  |  |  |  |
| Punjab | Ludhiana | 10.3 | 60.8 |
| Punjab | Sahibzada Ajit Singh | 19.2 | 67.0 |
| Punjab | Sangrur | 21.9 | 75.6 |
| Punjab | Amritsar | 26.6 | 44.2 |
| Punjab | Mansa | 27.9 | 53.3 |
| Punjab | Fatehgarh sahib | 31.1 | 62.7 |
| Punjab | Firozpur | 32.6 | 45.6 |
| Punjab | Patiala | 32.7 | 49.0 |
| Punjab | Hoshiarpur | 33.2 | 59.8 |
| Punjab | Bathinda | 34.5 | 44.0 |
| Punjab | Rupnagar | 34.5 | 69.5 |
| Punjab | Gurdaspur | 34.7 | 71.6 |
| Punjab | Shahid Bhagat Singh Nagar | 36.1 | 51.9 |
| Punjab | Tarn taran | 37.4 | 53.5 |
| Punjab | Moga | 42.0 | 50.5 |
| Punjab | Barnala | 45.7 | 50.3 |
| Punjab | Muktsar | 46.0 | 63.4 |
| Punjab | Jalandhar | 48.6 | 60.7 |
| Punjab | Faridkot | 50.4 | 60.8 |
| Punjab | Kapurthala | 57.3 | 67.3 |
|  |  |  |  |
| Rajasthan | Sirohi | 4.6 | 69.8 |
| Rajasthan | Barmer | 4.8 | 60.6 |
| Rajasthan | Rajsamand | 4.9 | 76.0 |
| Rajasthan | Kota | 6.3 | 73.7 |
| Rajasthan | Tonk | 6.4 | 75.3 |
| Rajasthan | Dungarpur | 6.7 | 76.4 |
| Rajasthan | Jaisalmer | 7.6 | 42.6 |
| Rajasthan | Bhilwara | 7.7 | 72.0 |
| Rajasthan | Udaipur | 8.6 | 79.1 |
| Rajasthan | Bikaner | 8.9 | 51.2 |
| Rajasthan | Pali | 8.9 | 53.7 |
| Rajasthan | Dhaulpur | 9.2 | 50.4 |
| Rajasthan | Sawai Madhopur | 9.2 | 49.0 |
| Rajasthan | Bharatpur | 10.4 | 56.7 |
| Rajasthan | Chittaurgarh | 11.1 | 71.4 |
| Rajasthan | Churu | 11.3 | 42.6 |
| Rajasthan | Dausa | 11.3 | 45.9 |
| Rajasthan | Ajmer | 11.5 | 69.0 |
| Rajasthan | Hanumangarh | 12.3 | 46.8 |
| Rajasthan | Alwar | 13.7 | 54.0 |
| Rajasthan | Sikar | 13.8 | 49.0 |
| Rajasthan | Karauli | 14.7 | 53.8 |
| Rajasthan | Bundi | 17.2 | 80.1 |
| Rajasthan | Jalor | 18.0 | 66.9 |
| Rajasthan | Nagaur | 18.3 | 51.9 |
| Rajasthan | Jaipur | 19.9 | 49.2 |
| Rajasthan | Banswara | 22.4 | 84.4 |
| Rajasthan | Jodhpur | 24.4 | 63.6 |
| Rajasthan | Ganganagar | 25.1 | 40.9 |
| Rajasthan | Pratapgarh | 25.4 | 75.1 |
| Rajasthan | Baran | 27.2 | 76.6 |
| Rajasthan | Jhunjhunun | 31.0 | 45.7 |
| Rajasthan | Jhalawar | 31.8 | 77.5 |
|  |  |  |  |
| Sikkim | East | 48.5 | 50.3 |
| Sikkim | South | 49.4 | 61.5 |
| Sikkim | North | 52.2 | 64.9 |
| Sikkim | West | 54.3 | 60.3 |
|  |  |  |  |
| Tamil Nadu | Kanniyakumari | 11.4 | 38.7 |
| Tamil Nadu | Tirunelveli | 18.5 | 62.2 |
| Tamil Nadu | Madurai | 21.3 | 53.6 |
| Tamil Nadu | Sivaganga | 23.6 | 52.4 |
| Tamil Nadu | Ramanathapuram | 24.2 | 48.6 |
| Tamil Nadu | Nagapattinam | 25.7 | 42.0 |
| Tamil Nadu | Kancheepuram | 26.0 | 45.5 |
| Tamil Nadu | Vellore | 31.8 | 50.3 |
| Tamil Nadu | Namakkal | 32.3 | 50.3 |
| Tamil Nadu | Tiruppur | 32.5 | 52.3 |
| Tamil Nadu | Thoothukkudi | 33.4 | 56.1 |
| Tamil Nadu | Coimbatore | 33.5 | 43.9 |
| Tamil Nadu | Thanjavur | 33.5 | 54.3 |
| Tamil Nadu | Thiruvallur | 33.9 | 49.9 |
| Tamil Nadu | Pudukkottai | 34.4 | 45.0 |
| Tamil Nadu | Cuddalore | 35.1 | 52.3 |
| Tamil Nadu | Chennai | 36.0 | 44.8 |
| Tamil Nadu | Dindigul | 36.4 | 43.2 |
| Tamil Nadu | Viluppuram | 37.0 | 56.8 |
| Tamil Nadu | Krishnagiri | 37.9 | 52.4 |
| Tamil Nadu | Perambalur | 38.3 | 56.4 |
| Tamil Nadu | Theni | 38.9 | 53.1 |
| Tamil Nadu | Tiruchirappalli | 39.9 | 60.0 |
| Tamil Nadu | Salem | 40.0 | 50.7 |
| Tamil Nadu | Karur | 41.0 | 53.3 |
| Tamil Nadu | Virudhunagar | 42.6 | 51.3 |
| Tamil Nadu | Erode | 42.7 | 51.1 |
| Tamil Nadu | Thiruvarur | 42.9 | 53.0 |
| Tamil Nadu | Ariyalur | 44.8 | 46.7 |
| Tamil Nadu | Dharmapuri | 44.8 | 58.1 |
| Tamil Nadu | Tiruvannamalai | 45.4 | 58.2 |
| Tamil Nadu | The Nilgiris | 46.9 | 52.6 |
|  |  |  |  |
| Tripura | North Tripura | 2.7 | 37.2 |
| Tripura | South Tripura | 6.9 | 50.4 |
| Tripura | Dhalai | 7.0 | 50.4 |
| Tripura | West Tripura | 11.0 | 51.8 |
|  |  |  |  |
| Uttar Pradesh | Shahjahanpur | 3.3 | 78.1 |
| Uttar Pradesh | Pilibhit | 3.6 | 78.5 |
| Uttar Pradesh | Jhansi | 3.7 | 77.2 |
| Uttar Pradesh | Banda | 3.9 | 62.4 |
| Uttar Pradesh | Moradabad | 4.7 | 74.6 |
| Uttar Pradesh | Muzaffarnagar | 4.8 | 79.4 |
| Uttar Pradesh | Aligarh | 4.8 | 67.0 |
| Uttar Pradesh | Saharanpur | 5.1 | 75.5 |
| Uttar Pradesh | Sant Ravidas Nagar | 5.1 | 63.9 |
| Uttar Pradesh | Mahamaya Nagar | 5.4 | 48.5 |
| Uttar Pradesh | Etawah | 5.5 | 39.6 |
| Uttar Pradesh | Bareilly | 5.5 | 74.3 |
| Uttar Pradesh | Mau | 5.6 | 62.1 |
| Uttar Pradesh | Farrukhabad | 6.0 | 39.2 |
| Uttar Pradesh | Fatehpur | 6.0 | 43.9 |
| Uttar Pradesh | Ballia | 6.0 | 60.7 |
| Uttar Pradesh | Sant Kabir Nagar | 6.2 | 68.9 |
| Uttar Pradesh | Ghaziabad | 6.2 | 62.0 |
| Uttar Pradesh | Mahoba | 6.4 | 78.4 |
| Uttar Pradesh | Ambedkar Nagar | 6.6 | 61.4 |
| Uttar Pradesh | Agra | 6.7 | 51.6 |
| Uttar Pradesh | Rampur | 7.2 | 77.3 |
| Uttar Pradesh | Kanshiram Nagar | 7.7 | 40.9 |
| Uttar Pradesh | Budaun | 7.8 | 59.2 |
| Uttar Pradesh | Firozabad | 7.9 | 46.9 |
| Uttar Pradesh | Bahraich | 8.2 | 73.9 |
| Uttar Pradesh | Shrawasti | 8.4 | 69.4 |
| Uttar Pradesh | Faizabad | 8.5 | 64.1 |
| Uttar Pradesh | Meerut | 9.0 | 72.7 |
| Uttar Pradesh | Jaunpur | 9.0 | 59.0 |
| Uttar Pradesh | Kanpur Nagar | 9.4 | 73.0 |
| Uttar Pradesh | Gautam Buddha Nagar | 10.1 | 68.8 |
| Uttar Pradesh | Baghpat | 10.3 | 79.1 |
| Uttar Pradesh | Mainpuri | 10.6 | 42.2 |
| Uttar Pradesh | Bara Banki | 10.8 | 44.0 |
| Uttar Pradesh | Gonda | 11.2 | 72.2 |
| Uttar Pradesh | Azamgarh | 11.2 | 61.6 |
| Uttar Pradesh | Siddharth Nagar | 11.3 | 65.1 |
| Uttar Pradesh | Varanasi | 11.4 | 58.9 |
| Uttar Pradesh | Etah | 11.6 | 40.5 |
| Uttar Pradesh | Mathura | 11.6 | 55.6 |
| Uttar Pradesh | Sitapur | 12.2 | 53.1 |
| Uttar Pradesh | Kheri | 13.2 | 49.9 |
| Uttar Pradesh | Balrampur | 13.4 | 73.0 |
| Uttar Pradesh | Lalitpur | 14.0 | 74.8 |
| Uttar Pradesh | Auraiya | 14.8 | 79.5 |
| Uttar Pradesh | Ghazipur | 15.4 | 68.5 |
| Uttar Pradesh | Lucknow | 15.5 | 71.5 |
| Uttar Pradesh | Kaushambi | 15.6 | 66.8 |
| Uttar Pradesh | Kannauj | 15.7 | 41.6 |
| Uttar Pradesh | Unnao | 16.1 | 44.7 |
| Uttar Pradesh | Gorakhpur | 16.7 | 60.2 |
| Uttar Pradesh | Basti | 17.0 | 71.2 |
| Uttar Pradesh | Pratapgarh | 18.2 | 61.9 |
| Uttar Pradesh | Rae Bareli | 18.2 | 60.8 |
| Uttar Pradesh | Sultanpur | 18.4 | 68.0 |
| Uttar Pradesh | Chitrakoot | 18.5 | 72.4 |
| Uttar Pradesh | Kanpur Dehat | 19.0 | 65.3 |
| Uttar Pradesh | Deoria | 19.5 | 68.5 |
| Uttar Pradesh | Bijnor | 19.7 | 72.5 |
| Uttar Pradesh | Jalaun | 22.2 | 84.5 |
| Uttar Pradesh | Jyotiba Phule Nagar | 25.4 | 75.4 |
| Uttar Pradesh | Allahabad | 26.3 | 61.3 |
| Uttar Pradesh | Hardoi | 26.4 | 45.1 |
| Uttar Pradesh | Bulandshahr | 28.1 | 66.0 |
| Uttar Pradesh | Hamirpur | 29.5 | 55.3 |
| Uttar Pradesh | Mahrajganj | 29.8 | 60.2 |
| Uttar Pradesh | Mirzapur | 30.3 | 62.6 |
| Uttar Pradesh | Sonbhadra | 32.3 | 58.7 |
| Uttar Pradesh | Chandauli | 33.3 | 66.8 |
| Uttar Pradesh | Kushinagar | 35.4 | 59.7 |
|  |  |  |  |
| Uttarakhand | Bageshwar | 5.4 | 49.5 |
| Uttarakhand | Chamoli | 6.2 | 53.2 |
| Uttarakhand | Pithoragarh | 6.4 | 41.8 |
| Uttarakhand | Almora | 11.6 | 49.3 |
| Uttarakhand | Hardwar | 11.8 | 71.1 |
| Uttarakhand | Champawat | 13.3 | 46.8 |
| Uttarakhand | Dehradun | 14.6 | 51.2 |
| Uttarakhand | Tehri Garhwal | 15.3 | 60.0 |
| Uttarakhand | Uttarkashi | 17.2 | 75.9 |
| Uttarakhand | Garhwal | 18.2 | 58.4 |
| Uttarakhand | Rudraprayag | 18.4 | 58.6 |
| Uttarakhand | Nainital | 18.4 | 58.6 |
| Uttarakhand | Udham Singh Nagar | 19.6 | 64.9 |
|  |  |  |  |
| West Bengal | Nadia | 14.3 | 36.3 |
| West Bengal | Darjiling | 16.6 | 45.6 |
| West Bengal | Maldah | 16.8 | 55.9 |
| West Bengal | Uttar Dinajpur | 21.0 | 64.0 |
| West Bengal | North Twenty Four Parganas | 21.0 | 53.7 |
| West Bengal | Hugli | 25.0 | 53.4 |
| West Bengal | Kochbihar | 25.4 | 58.6 |
| West Bengal | Birbhum | 29.4 | 58.6 |
| West Bengal | Murshidabad | 29.7 | 46.7 |
| West Bengal | Barddhaman | 30.1 | 45.2 |
| West Bengal | Kolkata | 30.2 | 69.3 |
| West Bengal | Paschim Medinipur | 30.4 | 54.9 |
| West Bengal | Jalpaiguri | 31.8 | 71.2 |
| West Bengal | Bankura | 32.2 | 47.0 |
| West Bengal | Haora | 32.9 | 58.4 |
| West Bengal | Puruliya | 33.2 | 66.3 |
| West Bengal | Purba Medinipur | 33.4 | 41.9 |
| West Bengal | South Twenty Four Parganas | 36.0 | 65.1 |
| West Bengal | Dakshin Dinajpur | 43.0 | 68.6 |
|  |  |  |  |
| Telangana | Mahbubnagar | 21.4 | 64.7 |
| Telangana | Hyderabad | 30.3 | 54.1 |
| Telangana | Khammam | 31.0 | 71.3 |
| Telangana | Rangareddy | 35.1 | 48.3 |
| Telangana | Karimnagar | 35.3 | 53.0 |
| Telangana | Medak | 36.2 | 67.4 |
| Telangana | Nizamabad | 37.3 | 66.2 |
| Telangana | Adilabad | 44.6 | 66.8 |
| Telangana | Warangal | 50.3 | 62.9 |
| Telangana | Nalgonda | 51.1 | 70.6 |
| *Note*: As of 2020, there were total 736 districts in India. However, the NFHS 2015-2016 was canvassed using the sampling frame based on Census 2011, and thus separate estimates for 92 newly formed districts were not available. | | | |
